# MicroRNA let-7b downregulates AML1-ETO oncogene expression in t(8;21) AML by targeting its 3′UTR

**DOI:** 10.1186/s40164-021-00204-7

**Published:** 2021-02-02

**Authors:** Daniel T. Johnson, Amanda G. Davis, Jie-Hua Zhou, Edward D. Ball, Dong-Er Zhang

**Affiliations:** 1 Moores Cancer Center, University of California, La Jolla, San Diego, CA USA; 2 Biological Sciences Graduate Program, University of California San Diego, La Jolla, San Diego, CA USA; 3 Division of Biological Sciences, University of California San Diego, La Jolla, San Diego, CA USA; 4 BMT Division, Department of Medicine, University of California San Diego, La Jolla, San Diego, CA USA; 5 Department of Pathology, University of California San Diego, La Jolla, San Diego, CA USA

**Keywords:** AML1-ETO (RUNX1-RUNX1T1), let-7, miRNA, t(8;21) acute myeloid leukemia

## Abstract

**Background:**

Acute myeloid leukemia (AML) with the t(8;21)(q22;q22) chromosomal translocation is among the most common subtypes of AML and produces the *AML1-ETO* (*RUNX1-ETO*, *RUNX1-RUNX1T1*) oncogenic fusion gene. AML1-ETO functions as an aberrant transcription factor which plays a key role in blocking normal hematopoiesis. Thus, the expression of AML1-ETO is critical to t(8;21) AML leukemogenesis and maintenance. Post-transcriptional regulation of gene expression is often mediated through interactions between *trans*-factors and *cis*-elements within transcript 3′-untranslated regions (UTR). *AML1-ETO* uses the 3′UTR of the *ETO* gene, which is not normally expressed in hematopoietic cells. Therefore, the mechanisms regulating AML1-ETO expression via the 3’UTR are attractive therapeutic targets.

**Methods:**

We used RNA-sequencing of t(8;21) patients and cell lines to examine the 3′UTR isoforms used by *AML1-ETO* transcripts. Using luciferase assay approaches, we test the relative contribution of 3′UTR *cis* elements to AML1-ETO expression. We further use let-7b microRNA mimics and anti-let-7b sponges for functional studies of t(8;21) AML cell lines.

**Results:**

In this study, we examine the regulation of AML1-ETO via the 3’UTR. We demonstrate that *AML1*-*ETO* transcripts primarily use a 3.7 kb isoform of the *ETO* 3′UTR in both t(8;21) patients and cell lines. We identify a negative regulatory element within the *AML1-ETO* 3′UTR. We further demonstrate that the let-7b microRNA directly represses AML1-ETO through this site. Finally, we find that let-7b inhibits the proliferation of t(8;21) AML cell lines, rescues expression of AML1-ETO target genes, and promotes differentiation.

**Conclusions:**

AML1-ETO is post-transcriptionally regulated by let-7b, which contributes to the leukemic phenotype of t(8;21) AML and may be important for t(8;21) leukemogenesis and maintenance.

## Introduction

The t(8;21)(q22;q22) translocation is among the most common chromosomal abnormalities in AML [1]. Although t(8;21) AML is categorized as a favorable-risk AML, there remains a high incidence of relapse (up to 56 % in some studies) and the median overall survival is only 5 years, highlighting the need for further studies and additional therapies [1–5]. The t(8;21) translocation results in a fusion of the *AML1* (*RUNX1*) locus on chromosome 21 with the *ETO* (*MTG8*, *RUNX1T1*) locus on chromosome 8, creating the *AML1-ETO* (*RUNX1-ETO, RUNX1-RUNX1T1*) fusion gene [6]. AML1 is an essential transcription factor for healthy hematopoiesis [7]. The N-terminal runt homology domain (RHD) of *AML1* mediates sequence-specific DNA-binding whereas the C-terminal transcriptional regulatory domain controls target gene transcription through the recruitment of essential co-factors [7]. ETO (RUNX1T1) is not normally expressed in hematopoietic progenitor cells [8–10], does not have DNA binding activity [11], and interacts with transcriptional co-repressors and co-activators [6, 11–16]. The *AML1-ETO* chimeric transcript contains the N-terminal RHD of *AML1* and nearly the entire *ETO* gene, including its 3′UTR [6]. Thus, the AML1-ETO fusion transcription factor impairs normal AML1-mediated myeloid differentiation through the transcriptional dysregulation of critical hematopoietic genes [6, 17–25], although additional mutations are also required for leukemogenesis [26, 27].

Stable expression of the *AML1-ETO* fusion gene is critical for both the initiation and maintenance of t(8;21) AML [28–34]. Transient knockdown of *AML1-ETO* induces differentiation [28, 30], inhibits proliferation [29, 30], and reduces leukemic burden [31]. AML1 expression is required for healthy hematopoiesis [7]. In contrast, ETO is not normally expressed in healthy hematopoietic progenitor cells [8–10]. Therefore, the factors which regulate AML1-ETO expression through the *ETO* portion of the chimeric transcript, including the 3’UTR, may be a unique sensitivity of t(8;21) leukemia cells compared to healthy hematopoietic cells.

The 3’UTR plays a crucial role in post-transcriptional gene expression by influencing the localization, stability, export, and translation efficiency of mRNA transcripts [35]. Sequence specific interactions between *cis*-elements within the 3′UTR and *trans*-factors, such as RNA binding proteins or microRNAs (miRNA), are major mediators of post-transcriptional regulation [35, 36]. miRNAs have also been shown to promote or suppress a variety of leukemic processes, including proliferation, apoptosis, differentiation, self-renewal, epigenetic regulation, and chemotherapy resistance [37–39]. The let-7 family of miRNAs are well described tumor suppressors in a variety of cancers, including leukemias [40–42]. Members of the let-7 miRNA family have demonstrated roles in sensitizing leukemic cells to chemotherapy [43–47]. Furthermore, the overexpression of let-7 miRNAs have also been shown to inhibit proliferation and promote differentiation in certain leukemic contexts [40, 48].

Since AML1-ETO expression is critical for t(8;21) AML leukemia, identifying factors that post-transcriptionally regulate AML1-ETO via the *ETO* 3′UTR may improve our understanding of how AML1-ETO expression is maintained and uncover suitable targets for t(8;21) AML therapy. Here, we demonstrate that *AML1-ETO* transcripts predominantly use the first 3.7 kb of the *ETO* 3′UTR, in both t(8;21) AML patients and cell lines. Using a luciferase assay approach to identify regulatory elements within the 3.7 kb *AML1-ETO* 3′UTR, we found a fragment of the *AML1-ETO* 3′UTR between 2.8 and 3.4 kb which was negatively regulated, increased expression upon inhibition of miRNA biogenesis, and contained a putative miRNA let-7 target site. We further showed that let-7b targets and represses AML1-ETO expression through this site. Finally, exogenous let-7b miRNA transfection inhibited the proliferation of t(8;21) AML cell lines, rescued expression of AML1-ETO downregulated target genes, and promoted leukemia cell differentiation. Our findings establish that the let-7b miRNA is a post-transcriptional regulator of *AML1-ETO and* affects the leukemic phenotype of t(8;21) AML.

## Methods

### Patients samples

t(8;21) AML samples were obtained from patients at UC San Diego Health. Following collection of peripheral blood or bone marrow from AML patients, cells were separated using Ficoll-Paque (VWR, Radnor, PA) and frozen until further use. Thawed products were diluted in 1x PBS supplemented with 1 mg/mL DNAse I (Sigma-Aldrich, St. Louis, MO) and washed with 1x PBS supplemented with 2 % FBS. Live, mononuclear cells were isolated by density gradient centrifugation using Ficoll-Paque (VWR). Magnetic bead CD34-enrichment was performed using a human CD34 MicroBead Kit (Miltenyi Biotec, Bergisch Gladbach, Germany), following the manufacturer’s instructions. An aliquot of CD34-enriched leukemic blasts was analyzed by flow cytometry to confirm that cells were > 95 % CD34^+^. RNA was extracted from patient blasts using Trizol reagent (ThermoFisher, Waltham, MA) according to the manufacturer’s instructions.

CD34^+^ HSPCs from healthy donors were obtained from Fred Hutchinson Cooperative Center for Excellence in Hematology (Seattle, Washington). Cells were thawed quickly and serially diluted with 1x PBS supplemented with 2 % FBS. Cells were resuspended in Trizol for RNA extraction.

### RNA-sequencing

Total RNA from Kasumi-1 and SKNO-1 cells was isolated using Trizol reagent (ThermoFisher). Library preparation of total RNA from Kasumi-1, SKNO-1, healthy HSPCs and patient blasts was performed using the TruSeq Stranded mRNA kit (Illumina, San Diego, CA) followed by sequencing on an Illumina HiSeq4000. Available public data for normal brain samples (SRR5938419 and SRR5938420) was downloaded from the NCBI Gene Expression Omnibus database [49]. Raw RNA-seq reads were aligned and mapped using HISAT2 using the usegalaxy public supercomputing platform [50].

### Transfection

One to two million cells of THP-1, HL-60, Kasumi-1, SKNO-1, or associated cell lines were resuspended with plasmid DNA or miRNA mimics in 100 µL of transfection buffer [140 mM Na_2_HPO_4_/NaH_2_PO_4_ (pH 7.2), 5 mM KCl, 15 mM MgCl_2_]. Cells were then transfected using programs P-19 (SKNO-1 and Kasumi-1), U-01 (THP-1), or Y-01 (HL-60) of the AMAXA II Nucleofector (Lonza; Basel, Switzerland) and were cultured in RPMI media plus 20 % FBS in a 37°C incubator with 5 % CO_2_. After 24 h the media was changed to RPMI plus 10 % FBS and 100 U/mL penicillin-streptomycin for the indicated times. For the HEK293T reporter assay, HEK293T cells were plated in 96-well plates and transfected with 5 ng of the psiCHECK2 3.7 kb AML1-ETO 3’UTR reporter plasmid DNA and 10 pmol of miRIDIAN™ microRNA Mimic negative control #2 or indicated miRIDIAN™ microRNA mimics (Dharmacon, Lafayette, CO), using DharmaFECT™ Duo transfection reagent (Dharmacon) according to manufacturer’s instructions.

### Proliferation assay

miRIDIAN™ microRNA Mimic negative control #2 or hsa-let-7b-5p (Dharmacon) were transfected into the SKNO-1 (100 pmol), THP-1 (100 pmol), HL-60 (200 pmol), or Kasumi-1 (200 pmol) cell lines. Viable cells were counted via trypan blue exclusion using a TC20 Automated Cell Counter (Bio-Rad Laboratories, Hercules, CA) on indicated days and were initially cultured at a density of 2 × 10^5^ cells per mL.

### Western blot

Primary antibodies included a mouse anti-α-tubulin (1:10,000) antibody (12G10, Developmental Studies Hybridoma Bank, University of Iowa, Iowa City, IA) and a previously described rabbit anti-AML1 (1:500) antibody generated by Covance[23]. Licor (Lincoln, NE) IRDye 680RD goat anti-mouse IgG and IRDye 800CW goat anti-rabbit IgG secondary antibodies (1:10,000) were used for visualization on a LI-COR Odyssey Classic imager. Image analysis and densitometry were performed using the LI-COR Application Software Version 3.0.

### Statistical analyses

All statistical analyses were performed using GraphPad Prism Software (Version 8.4.2). The specific tests used are documented in the corresponding figure legend. All t-tests were two-tailed. P values are denoted as follows: ns p > 0.05, *p < 0.05, **p < 0.01, ***p < 0.001.

## Results

### *AML1-ETO* transcripts primarily use the first 3.7 kb of the *ETO* 3′UTR in t(8;21) AML patients and cell lines

To uncover post-transcriptional regulators of the *AML1-ETO* oncogene that target the *ETO* 3’UTR, we first sought to identify which regions of the 3’UTR are present in *AML1-ETO* transcripts. Alternative polyadenylation regulates 3’UTR length and consequently the availability of *cis*-acting elements that bind *trans*-acting post-transcriptional regulators [35]. Therefore, to identify t(8;21) AML relevant interactions, we analyzed the endogenous *AML1-ETO* 3’UTR usage in t(8;21) AML patients and cell lines.

**Fig. 1:**
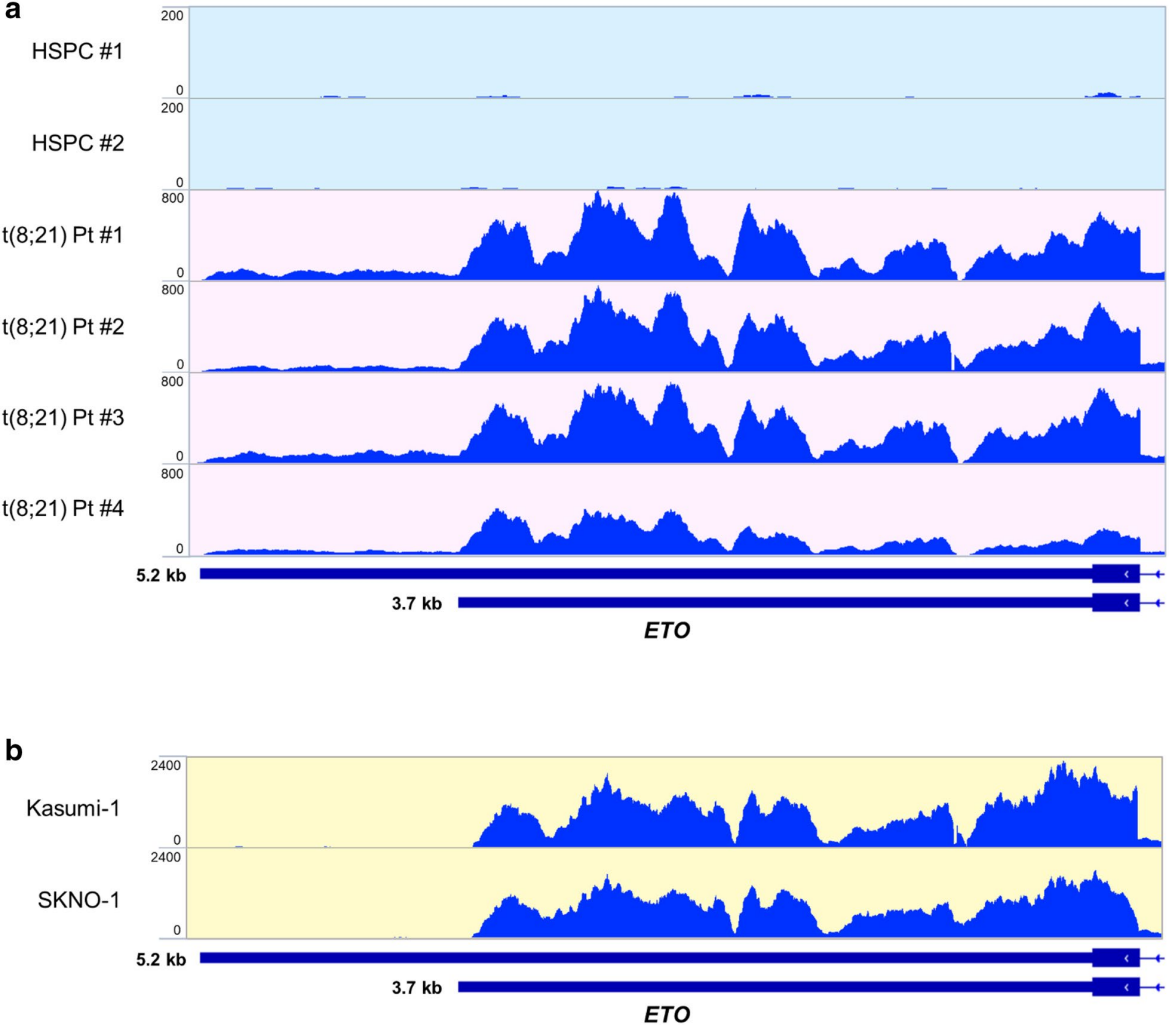
AML1-ETO transcripts primarily use a 3.7 kb isoform of the *ETO* 3′UTR in t(8;21) AML patients and cell lines. **a** Alignment of RNA-seq reads from healthy HSPCs (n = 2) and t(8;21) AML patient blasts (n = 4) to the final exon of *ETO*. Putative 3.7 kb and full-length 5.2 kb *ETO* 3′UTR isoforms are depicted below. **b** Alignment of RNA-seq reads from the t(8;21)^+^ Kasumi-1 and SKNO-1 cell lines to the final exon of *ETO*. Putative 3.7 kb and full-length 5.2 kb *ETO* 3′UTR isoforms are depicted below

We performed RNA-seq on peripheral blood derived CD34^+^ HSPCs from healthy donors and CD34^+^ enriched t(8;21) AML primary patients’ blasts. The longest predicted *AML1-ETO* 3′UTR sequence is 5.2 kb in length. However, analysis of the sequencing reads in the t(8;21) patients showed that the abundance of reads aligned within the first 3.7 kb of the *ETO* 3′UTR and only minor signal is detected up to the full 5.2 kb (Fig. 1a). Importantly, a validated poly(A) site is located at 3.7 kb, suggesting that *AML1-ETO* transcripts terminate at this proximal poly(A) site resulting in the observed shorter 3′UTR in patients. Consistent with previous reports [8–10], wild type *ETO* expression was undetectable in the healthy HSPC samples and the *ETO* RNA-seq reads in t(8;21) patient samples only map to the exons present in the *AML1-ETO* fusion (Fig. 1a, Additional file 1: S1a, b) [6]. To compare *AML1-ETO* 3′UTR usage to wild type *ETO* in a normal physiological context, we analyzed publicly available RNA-seq data of healthy brain cortex [49], which is known to express *ETO* [10]. The normal brain cortex RNA-seq reads included peaks in exons not involved in the *AML1-ETO* fusion and had a very similar pattern within the *ETO* 3′UTR, with the abundance of reads mapping within the first 3.7 kb of the *ETO* 3′UTR (Additional file 1: Fig. S1a, b). We further performed RNA-seq using the t(8;21) AML cell lines Kasumi-1 and SKNO-1. Interestingly, nearly all the *AML1*-*ETO* 3′UTR reads aligned within the first 3.7 kb in these cell lines (Fig. 1b). Taken together, our data suggests that *AML1-ETO* fusion transcripts primarily use the first 3.7 kb of the *ETO* 3′UTR in t(8;21) patients and cell lines.

### *AML1-ETO* 3′UTR *cis*-elements affect expression and are targeted by miRNAs

**Fig. 2:**
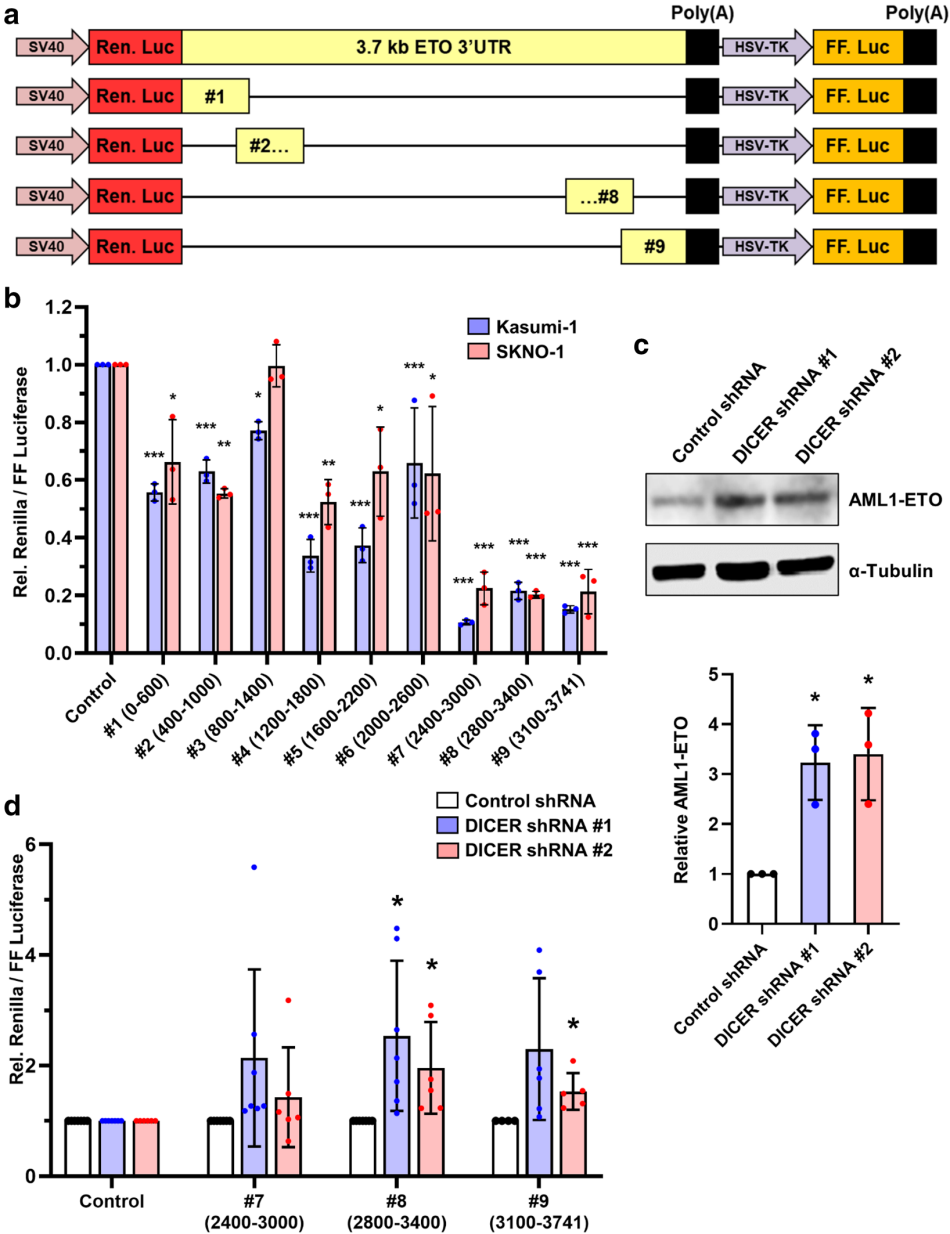
*AML1-ETO* 3′UTR *cis*-elements affect expression and are targeted by miRNAs. **a** Schematic of the dual luciferase reporters containing 600 bp fragments of the *AML1-ETO* 3′UTR. **b** Individual *AML1-ETO* 3′UTR dual luciferase reporters were transfected into Kasumi-1 and SKNO-1 cells and luciferase activity was measured. Assay results were normalized to an empty vector control. Values and error bars represent the average and SD of relative luciferase from three independent experiments. Significance is shown compared to empty vector control using one-way ANOVA with a post-hoc Tukey test. **c** Representative western blot and quantification of AML1-ETO protein in SKNO-1 DICER knockdown or control shRNA cell lines. Values and error bars represent the mean and SD of three independent experiments. **d** SKNO-1 cells expressing shRNAs targeting DICER or control shRNA were transfected with dual luciferase reporters expressing the indicated *AML1-ETO* 3′UTR fragments. Values and error bars represent the mean and SD of relative luciferase activity of shDICER compared to shCTRL from indicated number of independent experiments. Significance is shown compared to shCTRL control using unpaired t-tests corrected for multiple comparisons using the Holm-Sidak method

We next sought to identify the relevant regulatory sequences within the *AML1-ETO* 3′UTR. Because *AML1-ETO* patient transcripts predominantly used the first 3.7 kb of the *ETO* 3′UTR, we focused on sequences within this region. To identify regions within the 3’UTR that contain *cis*-elements that regulate *AML1-ETO* expression, we created a set of dual luciferase reporters that express *Renilla* luciferase with 3′UTRs containing progressive 600 bp fragments spanning the length of the first 3.7 kb of the *ETO* 3′UTR (with 200 bp overlaps between fragments) and firefly luciferase as an internal control (Fig. 2a). We mapped the effects of the *AML1-ETO* 3′UTR fragments on luciferase reporter expression compared to a control reporter through luciferase assays after transient transfection into the t(8;21)^+^ Kasumi-1 and SKNO-1 cell lines (Fig. 2b). The reporters containing *AML1-ETO* 3′UTR fragments had equal or lower expression compared to the control reporter, with some having up to 9-fold lower expression. We also observed up to 7-fold differences in luciferase activity between the reporters containing *AML1-ETO* 3′UTR fragments. Interestingly, reporters containing fragments near the 3’ end of the *AML1-ETO* 3′UTR (#7 2400–3000 bp, #8 2800–3400 bp, and #9 3100–3741 bp) had the lowest expression levels in both Kasumi-1 and SKNO-1 cells, suggesting that there are negatively regulated *cis*-elements in this region.

miRNAs are major mediators of negative post-transcriptional regulation. Normal miRNA biogenesis requires a multi-step process involving the RNAse DICER [51]. To assess the contribution of miRNAs in the regulation of AML1-ETO, we made stable shRNA-mediated DICER knockdown or control shRNA transduced SKNO-1 cell lines. We observed significantly increased levels of AML1-ETO protein upon DICER knockdown, compared with controls (Fig. 2c, Additional file 1: S2a). This result suggests that miRNAs regulate AML1-ETO protein levels and supports previous work that identified miR-193a, miR-9, and miR-29b as AML1-ETO regulators [52–54]. However, none of these miRNAs had predicted target sites within the 2600-3741 bp negatively regulated fragments near the end of the *AML1-ETO* 3’UTR [52–54]. To determine if miRNAs were involved specifically in the negative regulation of our *AML1-ETO* 3′UTR reporters, we transfected the lowest expressing 600 bp *AML1*-*ETO* 3′UTR reporters into the SKNO-1 shRNA control and shRNA DICER cell lines and performed luciferase assays (Fig. 2d). We observed significantly increased luciferase activity in both DICER knockdown cell lines transfected with the reporter containing the *AML1-ETO* 3’UTR fragment #8 (2800–3400 bp). Together, these results suggest that miRNAs are involved in the regulation of *AML1*-*ETO* and that at least one such miRNA specifically targets the *AML1*-*ETO* 3′UTR between 2800 and 3400 bp.

### let-7b targets and down regulates AML1-ETO via the 3’UTR

**Fig. 3:**
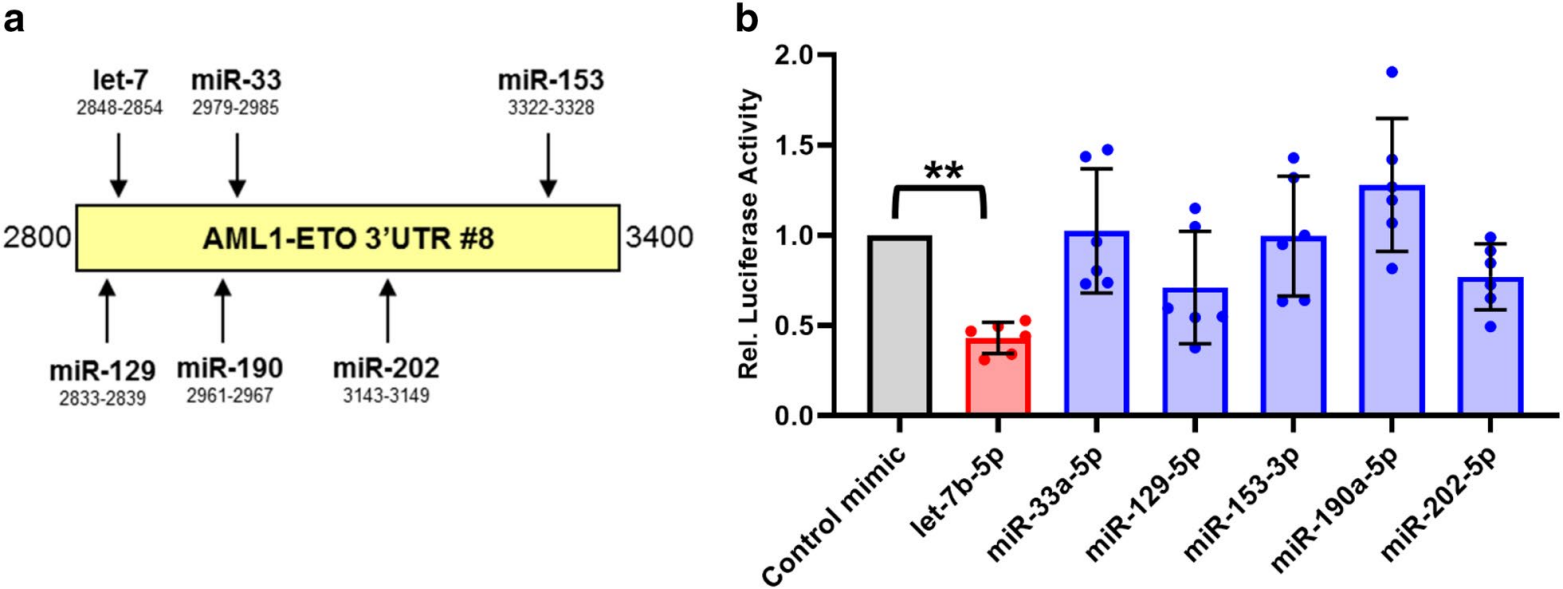
let-7b targets and down regulates an *AML1-ETO* 3’UTR reporter in HEK293T cells. **a** Schematic representation of miRNAs with predicted target sites within the 2800–3400 bp *AML1-ETO* 3′UTR fragment. Predictions were made using TargetScan 7.2. **b** miRNA mimics or non-targeting control mimic were co-transfected into HEK293T cells with the *AML1-ETO* 3.7 kb 3′UTR luciferase reporter and luciferase assays were performed 24 h post-transfection. Data is shown as relative luciferase activity normalized to non-targeting control mimic. Significance was determined using one-way ANOVA with a post-hoc Tukey test

To find miRNAs which putatively target the *AML1-ETO* 3’UTR between 2800 and 3400 bp, we used a miRNA targeting prediction model, TargetScan 7.2 [55]. We identified 6 families of miRNAs that were predicted to target this region: let-7, miR-33, miR-129, miR-153, miR-190, and miR-202 (Fig. 3a). We next tested the ability of all 6 candidate miRNA families to downregulate the AML1-ETO 3’UTR. We co-transfected miRNA “mimics” or non-targeting control mimics with a luciferase reporter containing the full 3.7 kb *AML1-ETO* 3’UTR in HEK293T cells (Fig. 3b). The let-7 family member mimic, let-7b, significantly decreased luciferase activity of the *AML1-ETO* 3′UTR reporter, suggesting that let-7b targets the *AML1-ETO* 3’UTR. Interestingly, among favorable-risk AML patients in the TCGA adult AML dataset [56], those with high expression of let-7b had significantly higher overall and disease-free survival (Additional file 1: Fig. S3a); though direct correlation between let-7b expression and overall and disease-free survival was only weakly positive (Additional file 1: Fig. S3b). Based on the potential survival benefit and the *AML1-ETO* 3’UTR reporter results, we focused on let-7b for our further studies.

**Fig. 4:**
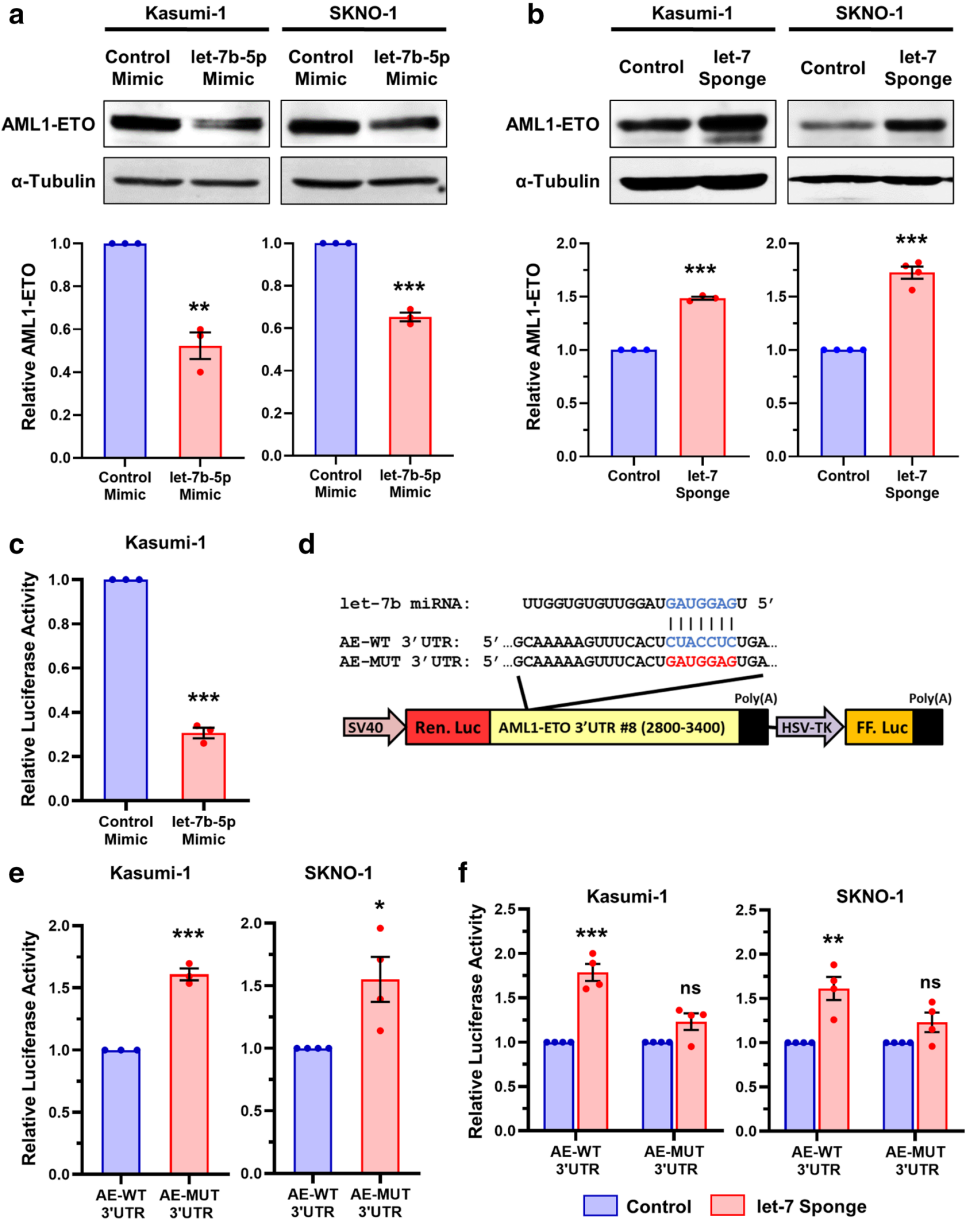
let-7b targets the 3’UTR and downregulates AML1-ETO in t(8;21) AML cells. **a**, **b** Representative western blots and protein quantification of Kasumi-1 and SKNO-1 whole cell lysates probed for endogenous AML1-ETO or α-Tubulin 48 h post-transfection with let-7b-5p or control miRNA mimics (**a**), or in cells stably expressing an anti-let-7 miRNA family sponge or control cells (**b**). **c** let-7b-5p or control miRNA mimics were co-transfected into Kasumi-1 cells with the *AML1-ETO* 3′UTR fragment #8 luciferase reporter and luciferase assays were performed 72 h post-transfection. Data is shown as relative luciferase activity normalized to control mimics. **d** Schematic of WT (AE-WT) and let-7 targeting mutant (AE-MUT) *AML1-ETO* 3′UTR fragment #8 luciferase reporters. **e** Luciferase assay results of AE-WT and AE-MUT reporters in Kasumi-1 or SKNO-1 cells 72 h post-transfection. **f** Kasumi-1 and SKNO-1 cell lines stably expressing either an anti-let-7 miRNA family sponge or control cells were transfected with the AE-WT or AE-MUT 3′UTR reporters and luciferase assays were performed 72 h later. Data is shown as relative luciferase activity normalized to control cell lines. All data in this figure is presented as the average and SD of the indicated number of individual experiments, unpaired t-tests with corrections for multiple tests using the Holm-Sidak method

We investigated the effect of let-7b on regulating endogenous AML1-ETO by transfecting let-7b-5p or non-targeting control miRNA mimics into the Kasumi-1 and SKNO-1 cell lines (Fig. 4a, Additional file 1: S4a). We observed significantly decreased AML1-ETO protein expression in both Kasumi-1 and SKNO-1 cells that were transfected with the let-7b-5p mimics compared to controls (Fig. 4a). To further test the role of let-7 miRNAs in regulating endogenous AML1-ETO expression, we made stable Kasumi-1 and SKNO-1 cell lines which expressed an anti-let-7 family member miRNA sponge [57]. Consistent with previous reports [58], we observed a modest decrease in mature let-7b-5p levels in the anti-let-7 miRNA sponge expressing cell lines because the major activity of bulged miRNA sponges is through miRNA decoy rather than miRNA decay (Additional file 1: Fig. S4b). Both the Kasumi-1 and SKNO-1 cell lines which stably expressed let-7 miRNA sponges showed significantly increased AML1-ETO protein levels compared to control cell lines (Fig. 4b). Together, these results demonstrate that AML1-ETO protein is down-regulated upon the addition of exogenous let-7b miRNA and is up-regulated when endogenous let-7 family members are inhibited.

The *AML1-ETO* 3’UTR contains a seed sequence for let-7 miRNA family members at 2.85 kb. Therefore, we next examined whether the regulation of AML1-ETO expression by let-7b was due to direct targeting of the *AML1-ETO* 3′UTR at this site. Indeed, luciferase activity of the *AML1-ETO* 3′UTR fragment #8 (2,800-3,400 bp) luciferase reporter was significantly decreased in Kasumi-1 cells co-transfected with let-7b-5p miRNA mimics compared to non-targeting control (Fig. 4c). Next, we introduced mutations of the putative let-7 target site (AE-MUT 3‘UTR) within the wild-type *AML1-ETO* 3’UTR #8 fragment luciferase reporter (AE-WT 3′UTR) (Fig. 4d). Luciferase assays in Kasumi-1 and SKNO-1 cells showed significantly increased activity in the reporters with the mutated let-7 target site compared to wild-type (Fig. 4e). Luciferase activity of the AE-WT 3′UTR reporter was also significantly greater in let-7 sponge expressing SKNO-1 and Kasumi-1 lines versus controls, whereas there was no significant difference in luciferase production by the AE-MUT 3′UTR reporter in these cellular contexts (Fig. 4f). Together, these results suggest that let-7b directly targets and down-regulates AML1-ETO via the 3′UTR.

### Expression of let-7b inhibits cell growth in t(8;21) AML cell lines

**Fig. 5:**
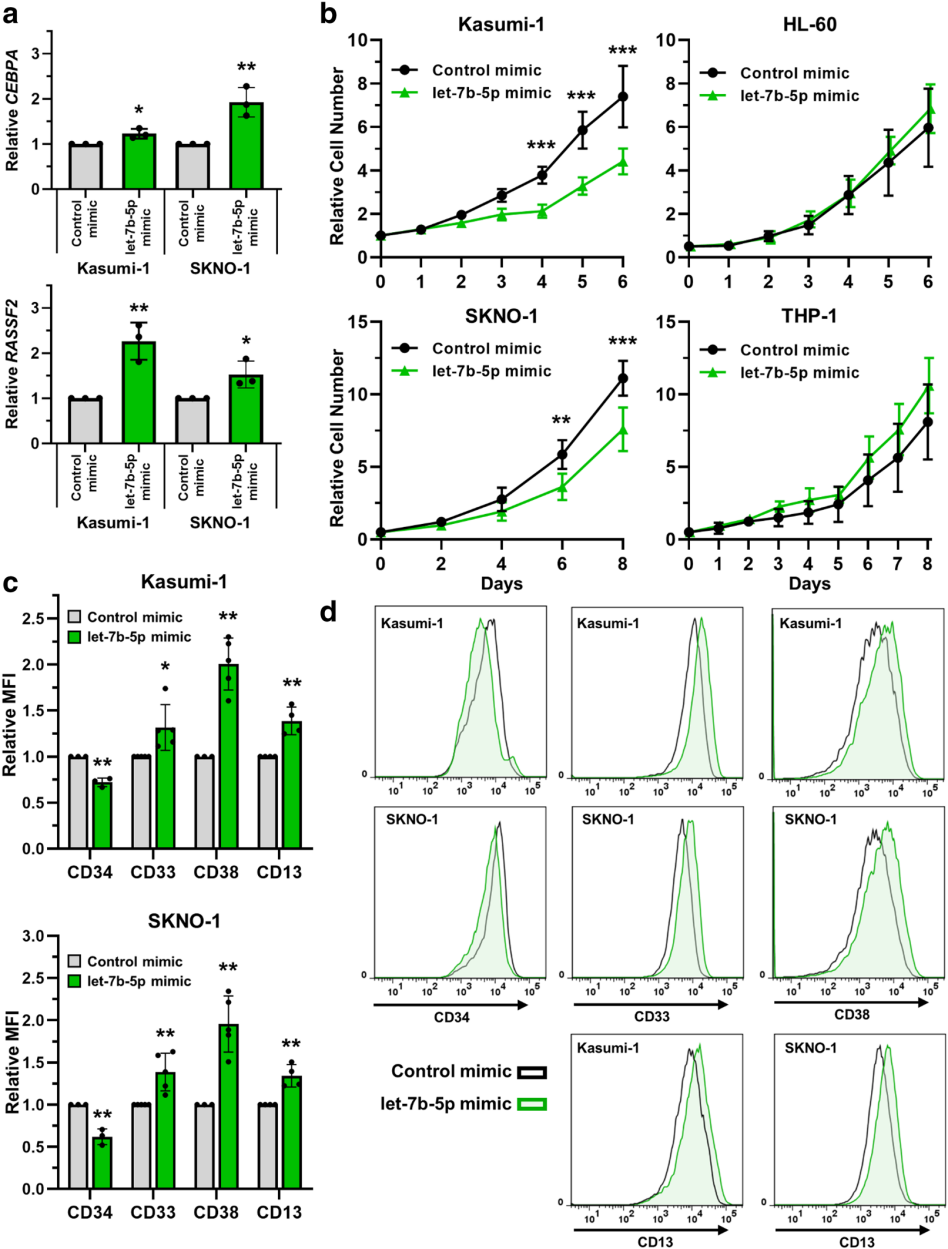
Expression of let-7b inhibits cell growth in t(8;21) AML cell lines. **a** Either let-7b-5p or control miRNA mimics were transfected into Kasumi-1 or SKNO-1 cells at an amount of 200 or 100 pmol for each respective cell line. Relative expression of *CEBPA* and *RASSF2* were determined by qPCR 96 h post-transfection. Data is presented as let-7b-5p mimic fold change relative to control. Values represent mean and SD of three individual experiments. Significance was determined by unpaired t-tests. **b** Cell growth of the t(8;21) AML cell lines Kasumi-1 and SKNO-1 or the non-t(8;21) AML cell lines HL-60 and THP-1, after transfection with let-7b-5p or control miRNA mimics, was determined by cell counting on the indicated days. Values represent mean and SD of four individual experiments. Significance was determined by unpaired t-tests of individual timepoints assuming homoscedasticity and with a correction for multiple tests using the Holm-Sidak method. **c**, **d** Relative mean fluorescent intensity (MFI) of CD34, CD33, CD38, and CD13 in Kasumi-1 or SKNO-1 96 h post-transfection with let-7b-5p or control miRNA mimics was determined using flow-cytometry. Values represent MFI relative to control mimic from indicated number of individual experiments. Significance determined by unpaired t-tests corrected for multiple comparisons using the Holm-Sidak method. **d** Representative histograms depict the fluorescence distribution of indicated markers in the indicated cell populations

Having observed that let-7b-5p overexpression can reduce AML1-ETO protein expression, we next wondered whether the degree of AML1-ETO downregulation has potential therapeutic relevance. Therefore, we first measured the expression of known transcriptionally repressed AML1-ETO target genes, *CEBPA* [17] and *RASSF2* [59], upon transfection of Kasumi-1 and SKNO-1 cells with the let-7b-5p miRNA mimic. In both cell lines, treatment with the miRNA mimic resulted in a significant increase of both transcripts in comparison to cells treated with a non-targeting control mimic (Fig. 5a). These results suggest that down regulation of AML1-ETO by let-7b-5p can partially rescue expression of genes that are transcriptionally repressed directly by AML1-ETO. We next measured the phenotypic impact of let-7 restoration on t(8;21)^+^ AML cell lines. The AML1-ETO protein has been shown to impair myeloid differentiation and transiently silencing its expression reduces cell proliferation and induces differentiation [24, 25, 28–30]. Likewise, the restoration of let-7 expression has been shown to inhibit cell proliferation and induce differentiation in cancers with low let-7 expression [60, 61]. Indeed, we observed significant inhibition of cell proliferation in the Kasumi-1 and SKNO-1 t(8;21) AML cell lines, but not in the HL-60 or THP-1 non-t(8;21) AML cell lines, after transfection with the let-7b-5p miRNA mimic versus the non-targeting control mimic (Fig. 5b). Additionally, both Kasumi-1 and SKNO-1 cells treated with the let-7b-5p mimic had significantly reduced cell surface expression of the hematopoietic stem cell marker, CD34 (Fig. 5 c, d). Furthermore, let-7b-5p mimic treated cells had significantly increased cell surface expression of the CD38 progenitor marker, as well as the CD33 and CD13 myeloid markers (Fig. 5 c, d). These results are indicative of a more differentiated cellular state, consistent with release of the AML1-ETO mediated myeloid differentiation block. Collectively, these results demonstrate that the restoration of let-7b levels rescues expression of repressed AML1-ETO target genes, impairs t(8;21) AML cell proliferation, and induces differentiation.

## Discussion

The AML1-ETO oncofusion protein impairs myeloid differentiation and is important for the initiation and maintenance of t(8;21) AML [24, 25, 28–34]. Thus, *AML1-ETO* 3′UTR is an appropriate target for t(8;21) AML therapies due to importance of AML1-ETO expression to t(8;21) AML and the lack of wild type *ETO* expression in healthy hematopoietic cells. However, the post-transcriptional regulation of AML1-ETO via its 3′UTR has not been well studied. We demonstrate that AML1-ETO transcripts primarily use a 3.7 kb isoform which is targeted and repressed by the let-7b miRNA. Furthermore, we show that the transient expression of let-7b in t(8;21) AML cell lines confers a tumor-suppressive phenotype, partially rescues AML1-ETO target gene expression, and induces differentiation.

Our study is the first examination of *AML1-ETO* 3′UTR usage in t(8;21) AML patients and cell lines. Interestingly, the 3′UTR usage within our set of four t(8;21) AML patient samples was quite similar, with the majority of RNA-seq reads mapping within the first 3.7 kb of the 3′UTR. Based on these results, further studies of factors that may regulate AML1-ETO via the 3′UTR should focus on the first 3.7 kb of the 3′UTR. The role of *AML1*-*ETO* 3′UTR isoform usage in t(8;21) leukemogenesis is difficult to determine because wild type *ETO* expression is undetectable in healthy HSPCs. However, future studies to examine how modulation of *AML1*-*ETO* 3′UTR usage affects AML1-ETO expression would be of interest and may lead to novel therapeutic strategies.

Our study further analyzed the contribution of different regulatory regions within the *AML1-ETO* 3′UTR to AML1-ETO expression. Our luciferase assay experiments using progressive *AML1-ETO* 3′UTR fragments showed up to 7-fold differences in luciferase activity between the fragments, with the lowest expression in fragments near the 3’ end of the 3.7 kb 3'UTR. These data suggest that the expression of AML1-ETO is controlled by negatively acting regulatory elements near the 3’ end of the 3.7 kb 3’UTR isoform. Strikingly, both the Kasumi-1 and SKNO-1 t(8;21) AML cell lines shared this trend, suggesting that these regulatory elements may be important in controlling the dosage of AML1-ETO for t(8;21) leukemic maintenance. Our DICER knockdown experiments showed a ~ 3-fold increase in AML1-ETO levels, which is consistent with other reported miRNA regulated genes [62, 63] and may be even greater than observed because DICER knockdown was incomplete. These results suggest that miRNAs are involved in the negative regulation of AML1-ETO, consistent with previous reports [52–54]. We further show that this miRNA-mediated regulation affects the *AML1-ETO* 3’UTR fragment between 2.8 and 3.4 kb. It is likely that additional *trans*-factors, such as RNA binding proteins, are also involved in the post-transcriptional regulation of *AML1-ETO*. Our luciferase reporter analysis of the *AML1*-*ETO* 3′UTR may be useful in further studies of *AML1-ETO* post-transcriptional regulation along this line.

Our study identifies let-7b as a direct regulator of the *AML1-ETO* oncogene. The let-7 family of miRNAs are well described tumor suppressors and are known to target several oncogenes including RAS, MYC and HMGA2 [61, 64–66]. Indeed, we found that overall and disease-free survival was significantly higher in let-7b high expression patients within the favorable-risk AML category, of which t(8;21) AML belongs, in the TCGA adult AML dataset [61]. We further demonstrate that transient expression of let-7b inhibits t(8;21) AML proliferation, rescues AML1-ETO target expression, and promotes differentiation. Although additional let-7b targets likely also contribute, these results agree with previous studies showing that directly silencing AML1-ETO with siRNA or shRNA releases the AML1-ETO mediated differentiation block of t(8;21) AML cells as evidenced by down-regulation of the hematopoietic stem cell marker CD34, and up-regulation of more mature myeloid markers such as CD38, CD33, and CD13 [28–30, 32, 33]. Identifying endogenous miRNAs that regulate AML1-ETO, such as let-7b, offers a potential method to silence AML1-ETO beyond the direct delivery of exogenous synthetic siRNAs. For example, pre-clinical strategies have been used to upregulate let-7 miRNA expression using small molecule inhibitors against the endogenous protein inhibitors of let-7 biogenesis, such as LIN28A/LIN28B [67–69], TUT4 [70, 71], or ADAR1 [72].

## Conclusions

Collectively, our study revealed the 3’UTR isoform usage of *AML1-ETO* in t(8;21) AML, examined regulatory regions throughout the *AML1-ETO* 3′UTR, and identified let-7b as a novel regulator of *AML1-ETO*. We further demonstrate that this regulation can inhibit t(8;21) AML cell line proliferation, partially reverse the AML1-ETO mediated differentiation block, and affect AML1-ETO transcriptional targets. Consequently, the post-transcriptional regulation of AML1-ETO through let-7b contributes to the leukemic phenotype of t(8;21) AML and may be important for t(8;21) leukemogenesis and maintenance.

## Supplementary Information

**Additional file 1.** Supplementary material and methods.

## Data Availability

RNA-sequencing (RNA-seq) data of patient samples, healthy, Kasumi-1, and SKNO-1 RNA-seq data was deposited at the European Nucleotide Archive, study accession number: PRJEB42786.

## Abbreviations

AML: Acute myeloid leukemia
HSPC: Hematopoietic stem and progenitor cell
miRNA: MicroRNA
RHD: Runt homology domain
UTR: Untranslated region
