# Supplementary material for: MicroRNA let-7b downregulates AML1-ETO oncogene expression in t(8;21) AML by targeting its 3′UTR
Source: Exp Hematol Oncol. 2021 Feb 2;10:8. doi: 10.1186/s40164-021-00204-7 (PMC7856722; doi:10.1186/s40164-021-00204-7)
Supplement: Supplementary file 1 — Additional file 1. Supplementary material and methods. [file 40164_2021_204_MOESM1_ESM.pdf]

SUPPLEMENTARY METHODS

Plasmids

MSCV-Puro (Clontech Laboratories, Mountain View, CA) was used as a control for let-7 sponge experiments. MSCV-Puro let-7 sponge was a gift from Phil Sharp (Addgene plasmid # 29766). For shRNA knockdown, control scrambled shRNA (gift from David Sabatini, Addgene plasmid #1864) and human DICER shRNA #1 and #2 constructs TRCN0000051260 and TRCN0000051261 were used. The psiCHECK-2 dual luciferase reporter vector (Promega, Madison, WI) was utilized for all luciferase assays, and all 3'UTR sequences were subcloned into the Xho1 and Not1 sites. The full-length 5.2 kb ETO 3'UTR was amplified from KG-1a (ATCC CCL-246.1) genomic DNA using following primer pairs: forward (5'– ACGTGAACTCAGAACTGTCGGAG –3'), reverse (5'– CATGATTAGGCAAACACAAC –3'). All canonical poly-adenylation sites (AATAAA) were removed by overlap PCR. The series of 600 bp *ETO* 3'UTR fragments were amplified from the full-length 5.2 kb plasmid using the listed primers (**Table S1**). The psiCHECK-2 3.7kb AML1-ETO 3'UTR reporter was cloned from the full-length 5.2 kb plasmid using the ETO_3'UTR_#1 forward and ETO_3'UTR_#9 Reverse primers (**Table S1**). The let-7-5p seed sequence mutant 3'UTR reporters were created through overlap PCR using the listed primers (**Table S1**).

Cell Culture

Human AML cell lines Kasumi-1, SKNO-1, THP-1, and HL-60 were cultured in RPMI media supplemented with 10% FBS and 100U/mL PSQ. Although SKNO-1 cells were initially established as a GM-CSF-dependent cell line [1], over time in culture, they have lost their cytokine dependence. HEK293T cells were cultured in DMEM media supplemented with 10% FCS and 100U/mL PSQ. All cells were maintained in a 37°C incubator with 5% CO_2.

Viral Transduction

For retroviral transduction experiments, retrovirus was produced in HEK293T cells. Transfections of HEK293T cells were conducted by combining 5 μg of MSCV-Puro or MSCV-puro let-7 vectors, 5 μg of packaging vector (pCL-10A1), and 40 μl of polyethylenimine (PEI) in 1 mL of Opti-MEM reduced serum medium (Thermo Fisher, Waltham, MA). Approximately 16 h post-transfection, media was aspirated, cells were washed once in PBS, and 7 mL of fresh RPMI media supplemented with 10% fetal bovine serum and 1% penicillin-streptomycin was added to each plate. 24 h after the media change, RPMI media containing retroviral particles was collected, passed through a 0.45 μm syringe filter, pooled, and supplemented with polybrene (4 μg/mL). Kasumi-1 or SKNO-1 cells were resuspended in retroviral media at a concentration of approximately 0.5 x 10^6cells/mL and were transduced in 6-well plates by centrifugation (2,000 x g) for 3 h at 32°C in an Allegra X-12R centrifuge (Beckman Coulter, Brea, CA) on two consecutive days. 24 h following the second transduction, cells were resuspended in fresh RPMI media supplemented with 1 μg/mL puromycin. After 48 h of puromycin selection, cells were diluted and maintained at 0.5 μg/mL puromycin until future analysis.

For DICER shRNA knockdown experiments, lentiviral transduction of shRNA constructs was performed. Transfection of HEK293T cells was conducted by combining 5 μg of psPAX2, 2.5 μg of pMD2.G, and 3 μg of respective pLKO.1-based shRNA vector, and 44 μl of polyethylenimine (PEI) in 1 mL of Opti-MEM reduced serum medium (Thermo Fisher, Waltham, MA). Approximately 16 h post-transfection, media was aspirated, cells were washed once in PBS, and 7 mL fresh RPMI supplemented with 10% fetal bovine serum and 1% penicillin-streptomycin was added to each plate. 24 h following media change, RPMI media containing lentiviral particles was collected, passed through a 0.45 μm syringe filter, pooled, and supplemented with polybrene (final working concentration: 4 μg/mL). Kasumi-1 or SKNO-1 cells were resuspended in lentiviral media at a concentration of approximately 0.5 x 10^6cells/mL and were transduced in 6-well plates by centrifugation (2,000 x g) for 3 h at 32°C in an Allegra X-12R centrifuge (Beckman Coulter, Brea, CA). 24 h following the transduction, cells were

resuspended in fresh RPMI media supplemented with 1 µg/mL puromycin. After 48 h of puromycin selection, cells were diluted and maintained at 0.5 µg/mL puromycin until future analysis.

Luciferase Assays

For the 3'UTR 600 bp-scanning due luciferase reporter assay, 100 ng and 500 ng plasmid DNA were transfected into Kasumi-1 cells and SKNO-1 cells, respectively, and cell lysates were prepared 48 h later. For luciferase assays in SKNO-1 shRNA experiments, 0.5-1 µg of control luciferase reporter DNA was transfected in each sample and cell lysates were prepared 72 h later. For the miRNA mimic luciferase assays, Kasumi-1 cells were transfected with 100 ng of luciferase reporter plasmid DNA, containing the 600 bp *ETO* 3'UTR fragment #8, and 100 pmol of negative control or hsa-let-7b-5p miRNA mimics; cell lysates were prepared 72 h later. For let-7 target site mutation luciferase assays experiments, Kasumi-1 or SKNO-1 cells were transfected with 0.5 or 2 µg of luciferase reporter plasmid DNA, containing the 600 bp *ETO* 3'UTR fragment #8 with or without let-7 target site mutation, and cell lysates were prepared 48 h later. For the let-7 sponge experiments, MSCV-Puro let-7 sponge and control MSCV-Puro expressing Kasumi-1 or SKNO-1 cell lines were transfected with 0.5 or 1 µg of luciferase reporter plasmid DNA, containing the 600 bp *ETO* 3'UTR fragment #8 with or without let-7 target site mutation, and cell lysates were prepared 48 h later. The Dual-Luciferase Reporter Assay System (Promega, Madison, WI) was used to make all cell lysates and firefly/*renilla* luciferase activities were measured using a Monolight 3010 luminometer (BD Biosciences, San Jose, CA) following manufacturer's instructions.

qPCR Analysis

For miRNA-qPCR, isolation of total RNA (including small RNAs) was performed using the mirVana™ miRNA isolation Kit (Thermo Fisher, Waltham, MA) according to the manufacturer's instructions. Universal polyadenylation and reverse transcription of miRNAs within total RNA was done using the miRCURY LNA RT system (Qiagen, Venlo, Holland) according to the manufacturer's instructions. Quantification of specific mature miRNAs was performed using technical triplicate miRCURY LNA

miRNA PCR assays for hsa-let-7b-5p and hsa-miR-103a-3p with the miRCURY LNA SYBR Green PCR Kit (Qiagen, Venlo, Holland) run on the CFX Connect Real-Time PCR Detection System (Bio-Rad Laboratories, Hercules, CA) following the manufacturer's instructions. Data analysis was performed using a standard delta-delta Ct method relative to the geometric-mean of hsa-miR-103a-3p.

For qPCR of *RASSF2* and *CEBPA*, isolation of total RNA (including small RNAs) was performed using the mirVana™ miRNA isolation Kit (Thermo Fisher, Waltham, MA) according to the manufacturer's instructions. For qPCR of *DICER*, cell lysis and RNA isolation were performed using Trizol reagent (Thermo Fisher, Waltham, MA) according to the manufacturer's instructions. cDNA preparation and quantitative PCR was performed as previously described [2], using the listed primers (Table S1). Data analysis was performed using a standard delta-delta Ct method relative to the geometric-mean of the *GAPDH* reference gene.

TCGA clinical and miRNA-sequencing dataset

Clinical information and pre-processed TCGA miRNA-sequencing data from AML patients in the favorable risk category (n = 36) was obtained through the TCGA data portal (https://tcga-data.nci.nih.gov/tcga) [3]. We used the "reads per million miRNA mapped" (RPMM) from the TCGA LAML miRNA quantification files as the miRNA expression values.

Flow Cytometry

Cells were stained with 7AAD (BD Biosciences, San Jose, CA) and PE-conjugated antibody against human CD34 (Miltenyi Biotec, AC136, Bergisch Gladbach, Germany), APC/Cy7-conjugated human CD13 (Biolegend, WM15, San Diego, CA), PE/Cy7-conjugated human CD33 (eBioscience, WM-53, San Diego, CA) or PE/Cy7-conjugated CD38 (Biolegend, HIT2) for flow cytometric analysis. Cells were analyzed with a FACS Canto cytometer (BD Biosciences, San Jose, CA), non-viable 7AAD+ cells were excluded.

SUPPLEMANTARY REFERENCES

1. Matozaki S, Nakagawa T, Kawaguchi R, Aozaki R, Tsutsumi M, Murayama T, et al. Establishment of a myeloid leukaemic cell line (SKNO-1) from a patient with t(8;21) who acquired monosomy 17 during disease progression. Br J Haematol. 1995;89(4):805-11.
2. Stoner SA, Yan M, Liu KTH, Arimoto KI, Shima T, Wang HY, et al. Hippo kinase loss contributes to del(20q) hematologic malignancies through chronic innate immune activation. Blood. 2019;134(20):1730-44.
3. Ley TJ, Miller C, Ding L, Raphael BJ, Mungall AJ, Robertson AG, et al. Genomic and Epigenomic Landscapes of Adult De Novo Acute Myeloid Leukemia. New Engl J Med. 2013;368(22):2059-74.

Supplementary Table 1. Primers used in this study.

Primer Name	Sequence 5'->3'	Purpose
ETO_3'UTR_#1_0-600-FWD-XhoI	TTTCGCTCGAGACGTGAACTCAGAACTGTCGGAG	Cloning into psiCheck2
ETO_3'UTR_#1_0-600-REV-NotI	TTTTTAGCGGCCGCACCGAACATCTGTGTCTCCTTCC	Cloning into psiCheck2
ETO_3'UTR_#2_400-1000-FWD-XhoI	TCCTCCTCGAGATATTAAAAGTCACAATGTTCTTTA	Cloning into psiCheck2
ETO_3'UTR_#2_400-1000-REV-NotI	TCTCTCGCGGCCGCATTTTTAAAATTATTTTTTTCAATAT	Cloning into psiCheck2
ETO_3'UTR_#3_800-1400-FWD-XhoI	TCCTCCTCGAGACACACACACACACACACACACACACACAAGTAAGAGACTCAGCCTGC	Cloning into psiCheck2
ETO_3'UTR_#3_800-1400-REV-NotI	ACGCTCGCGGCCGCCATGCTGGTTTTCAGGAAAAAAAAAAAATCCTATAAGAG	Cloning into psiCheck2
ETO_3'UTR_#4_1200-1800-FWD-XhoI	TTCGCCTCGAGAGTTGGTGAGAAACCTGTATC	Cloning into psiCheck2
ETO_3'UTR_#4_1200-1800-REV-NotI	TTTTAAGCGGCCGCAATACTGTATAACCTGGCATTC	Cloning into psiCheck2
ETO_3'UTR_#5_1600-2200-FWD-XhoI	TTTGGCTCGAGTTTTTTAAATCATTATTAGGGAC	Cloning into psiCheck2
ETO_3'UTR_#5_1600-2200-REV-NotI	TACTGTGCGGCCGCGATACTTAAAAGCCACTTAAAACTG	Cloning into psiCheck2
ETO_3'UTR_#6_2000-2600-FWD-XhoI	TTTGGCTCGAGGTTGTGTGCTTTAGGAAAGTCAGC	Cloning into psiCheck2
ETO_3'UTR_#6_2000-2600-REV-NotI	TTTTTTGCGGCCGCTGGGTATGACATACCTCATTTTTGGG	Cloning into psiCheck2
ETO_3'UTR_#7_2400-3000-FWD-XhoI	TTAGACTCGAGACCTGCTTAAAGTGAAATGAAAG	Cloning into psiCheck2
ETO_3'UTR_#7_2400-3000-REV-NotI	ACGATCGCGGCCGCGTTTATGAGGTCTGCATTGTTACC	Cloning into psiCheck2
ETO_3'UTR_#8_2800-3400-FWD-XhoI	TTAGACTCGAGTCCTCTCCTCCTGTTTTGCTACATTCTCCTCAGTGGC	Cloning into psiCheck2
ETO_3'UTR_#8_2800-3400-REV-NotI	ACGATCGCGGCCGCTGTCCCTTGAGGAATTCGGACAACATGG	Cloning into psiCheck2
ETO_3'UTR_#9_3145-3747-FWD-XhoI	TTAGACTCGAGGATAGGAATAGGGCGTCCTCT	Cloning into psiCheck2
ETO_3'UTR_#9_3145-3747-REV-NotI	ACGATCGCGGCCGCTTTTTTTCAACTTTACACAGTAAAGAATACAACAATACC	Cloning into psiCheck2
ETO_3'UTR_let7MUT_overlap-FWD	CAGTGGCAAAAAGTTTCACTGATGGAGTGACAGCATGTATATTGCAC	Overlap PCR
ETO_3'UTR_let7MUT_overlap-REV	GTGCAATATACATGCTGTCACTCCATCAGTGAAACTTTTTGCCACTG	Overlap PCR
hRASSF2_qPCR_F1	CAGGAGGAAGAAGATCGGGAAGTAA	qPCR Primer
hRASSF2_qPCR_R2	GAAAGAAAGTGCCTAGCTTCCTGG	qPCR Primer
hCEBPA_qPCR_F1	AACCTTGTGCCTTGGAAATG	qPCR Primer
hCEBPA_qPCR_R1	CCCTATGTTTCCACCCCTTT	qPCR Primer
hGAPDH_qPCR_F1	TCGCTCAGACACCATGGGGAAG	qPCR Primer
hGAPDH_qPCR_R1	GCCTTGACGGTGCCATGGAATTTG	qPCR Primer
hDICER1_qPCR_F1	AAAGCCAAATGGGAAAGTCTGC	qPCR Primer
hDICER1_qPCR_R1	AAGGCAGTGAAGGCGATAAAGTAT	qPCR Primer

Supplementary Figure 1. Additional RNA-seq Alignments

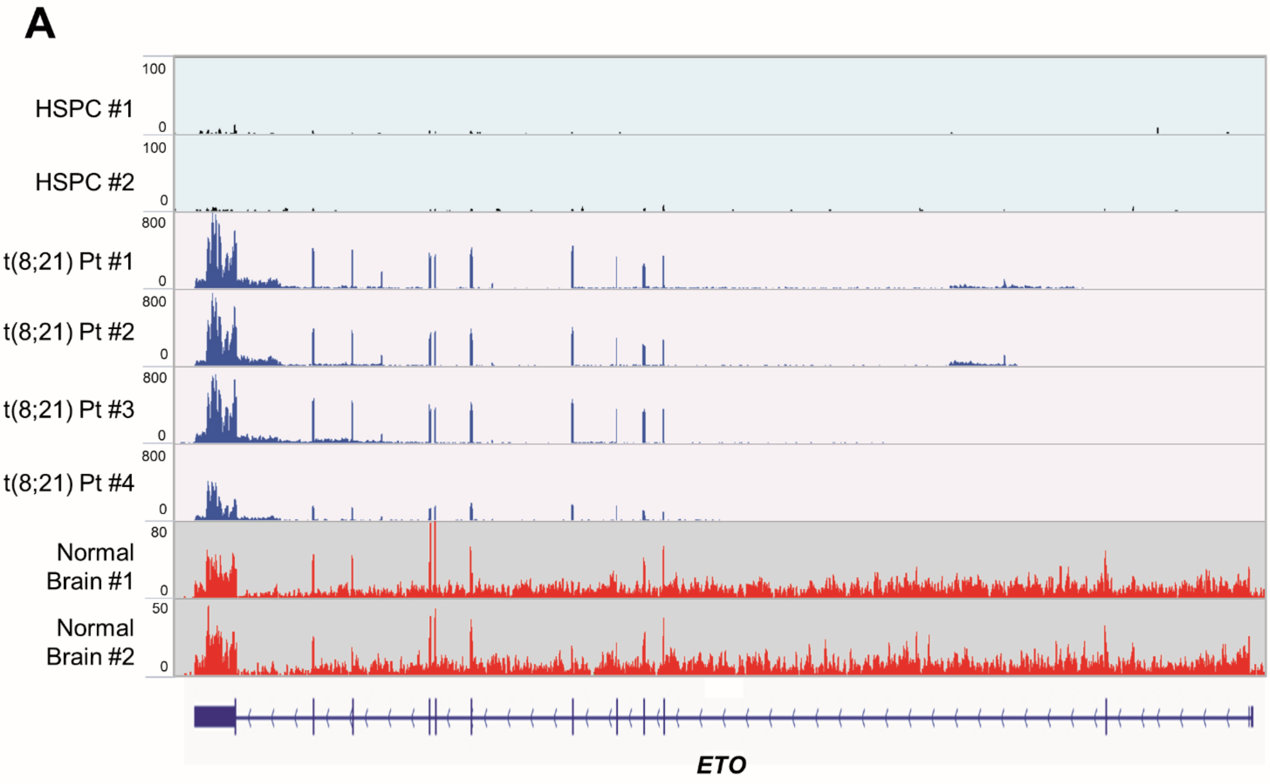

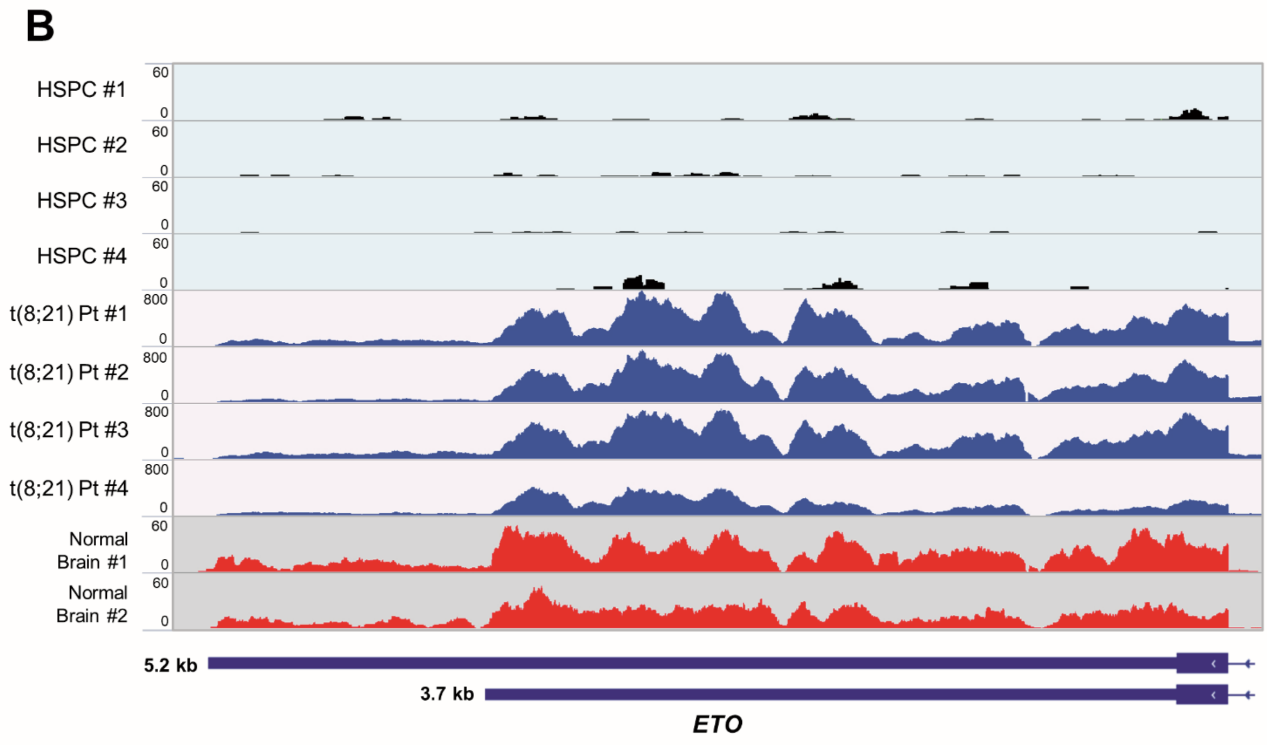

Supplementary Figure 2. *DICER* knockdown in SKNO-1 shRNA lines

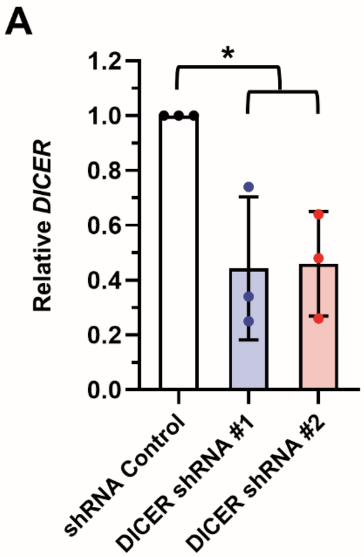

Supplementary Figure 3. Correlation of survival and let-7b expression in favorable-risk AML

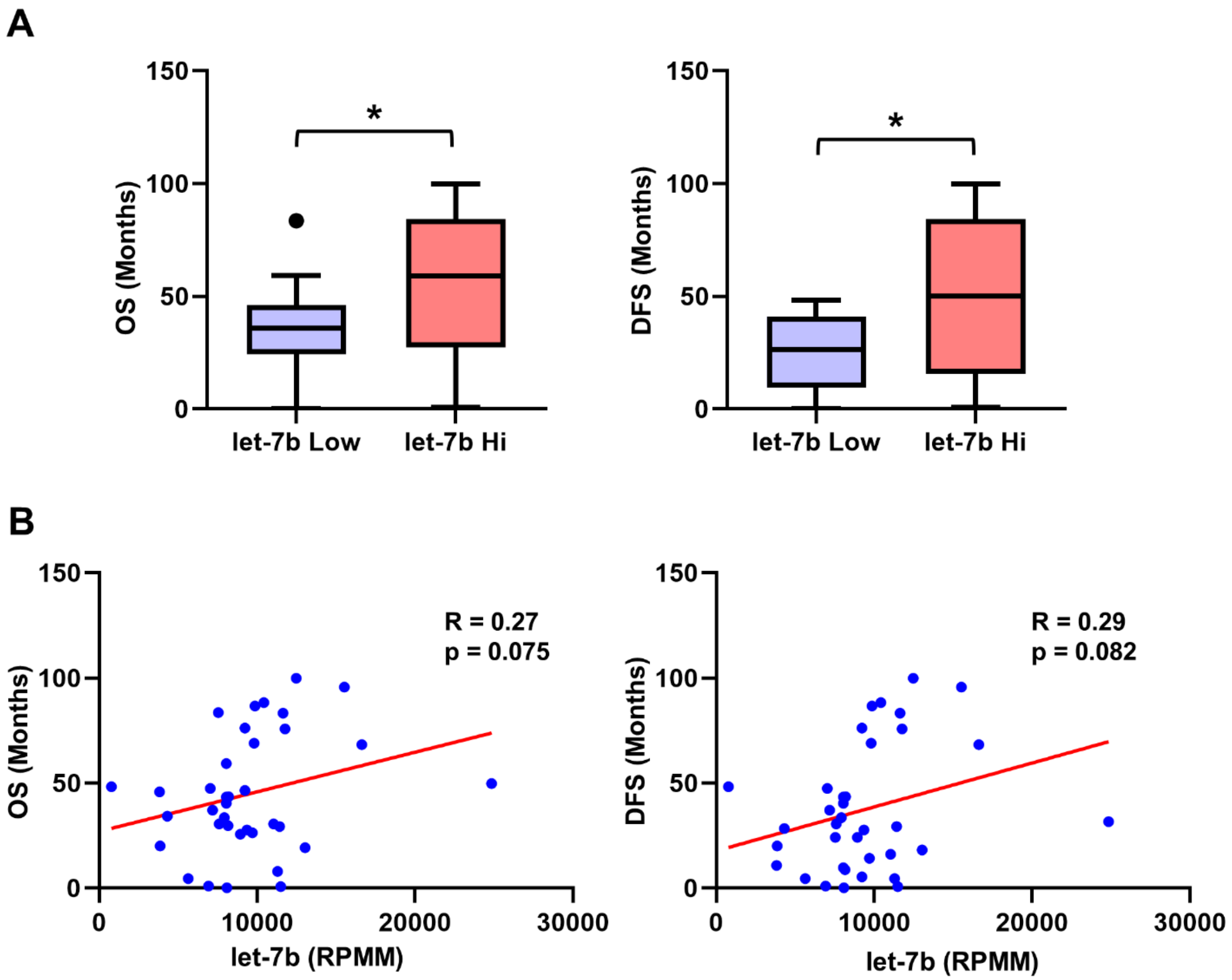

Supplementary Figure 4. Quantification of mature let-7b-5p change in associated experiments

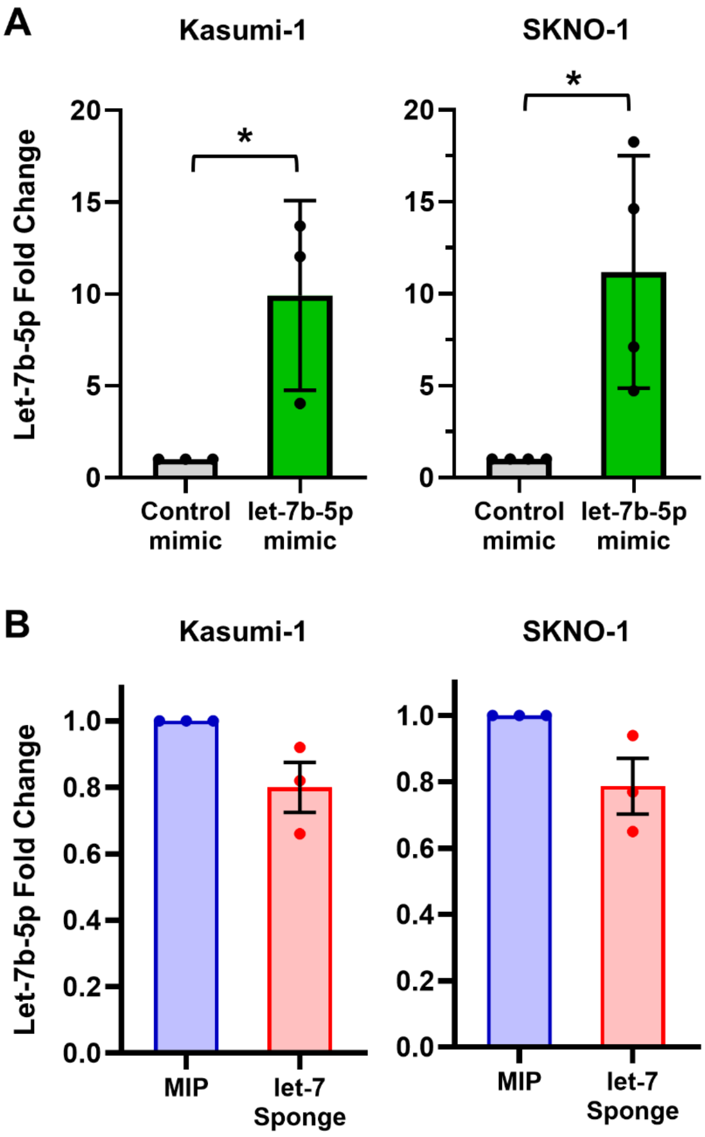

SUPPLEMENTARY FIGURE LEGENDS

Supplementary Figure 1. Additional RNA-seq alignments. (A) Alignment of RNA-seq reads from healthy HSPCs (n = 2), t(8;21) AML patient blasts (n = 4), and public normal human brain data (n = 2) (SRR5938419 and SRR5938420) to the full *ETO* gene. Exons 1 and 2 are not involved in *AML1-ETO* translocation. (B) Alignment of RNA-seq reads from all healthy HSPCs (n = 4), t(8;21) AML patient blasts (n = 4), and public normal human brain data (n = 2) (SRR5938419 and SRR5938420) to the final exon of *ETO*. Putative 3.7 kb and full-length 5.2 kb *ETO* 3'UTR isoforms are depicted below.

Supplementary Figure 2. *DICER* knockdown in SKNO-1 shRNA cell lines. (A) qPCR quantification of relative *DICER* expression in SKNO-1 shRNA cell lines from indicated number of independent experiments. p values indicated from unpaired t-test (* $p < 0.05$).

Supplementary Figure 3. Correlation of survival and let-7b expression in favorable-risk AML. (A) Overall survival (OS) and disease-free survival (DFS) of low (bottom 50%, n = 18) or high (top 50%, n = 18) let-7b expressing patients among favorable-risk AML samples in the TCGA cohort of adult AML samples. Significance determined by unpaired t-test. (B) Correlation of OS or DFS and let-7b normalized read counts per million mapped (RPMM) among favorable-risk AML samples in the TCGA cohort. Linear regression line is shown (red). Correlation determined using Pearson's correlation coefficient.

Supplementary Figure 4. Quantification of mature let-7b-5p change in associated experiments. (A) Kasumi-1 and SKNO-1 cell lines were treated with 200 or 100 pmol, respectively, of let-7b-5p or control miRNA mimics. Relative let-7b-5p expression levels compared to endogenous control miR-103a-3p were determined using microRNA qPCR. Data is presented as let-7b-5p fold change relative to control mimic transfection. (B) Relative let-7b-5p expression levels were determined using lysates from Kasumi-1 and SKNO-1 cell lines stably expressing an anti-let-7 miRNA family sponge or controls.

Data is shown as let-7b-5p fold change relative to control lines. All data is presented as the average and SD of indicated number of individual experiments, unpaired t-test (* $p < 0.05$).
